# Multiple ultrasound cavitation-enabled treatments for myocardial reduction

**DOI:** 10.1186/s40349-017-0107-x

**Published:** 2017-11-09

**Authors:** Douglas L. Miller, Xiaofang Lu, Chunyan Dou, Yiying I. Zhu, Mario L. Fabiilli, Gabe E. Owens, Oliver D. Kripfgans

**Affiliations:** 1 Department of Radiology, University of Michigan School of Medicine, Ann Arbor, MI 48109 USA; 2 Department of Pediatrics (Cardiology), University of Michigan School of Medicine, Ann Arbor, MI 48109 USA; 3 University of Michigan School of Medicine, 3240A Medical Sciences Building I, 1301 Catherine Street, Ann Arbor, MI 48109-5667 USA

**Keywords:** Myocardial contrast echocardiography, Arrhythmia, Cardiac myocyte necrosis, Hypertrophic cardiomyopathy

## Abstract

**Background:**

Ultrasound myocardial cavitation enabled treatment (MCET) is an image-guided method for tissue reduction. In this study, a strategy of fractionated (multiple) treatments was tested for efficacy.

**Methods:**

Dahl SS rats were anesthetized and prepared for treatment with a focused ultrasound transducer in a warm water bath. Aiming at the anterior left ventricular wall was facilitated by imaging with a 10 MHz phased array (10S, GE Vivid 7, GE Vingmed Ultrasound, Horten, Norway). MCET was accomplished at 1.5 MHz by pulse bursts of 4 MPa peak rarefactional pressure amplitude, which were intermittently triggered 1:8 from the ECG during infusion of a microbubble suspension for cavitation nucleation. Test groups were sham, a 200 s treatment, three 200 s treatments a week apart, and a 600 s treatment. Treatment outcome was observed by plasma troponin after 4 h, echocardiographic monitoring and histology at 6 wk.

**Results:**

The impacts of the fractionated treatments summed to approximately the same as the long treatment; e. g. the troponin result was 10.5 ± 3.2 for 200 s, 22.7 ± 5.4 (*p* < 0.001) for the summed fractionated treatments and 29.9 ± 6.4 for 600 s (*p* = 0.06 relative to the summed fractionated). While wall thickness was not reduced for the fractionated treatment, tissue strain was reduced by 35% in the target area relative sham (*p* < 0.001).

**Conclusion:**

The ability to fractionate treatment may be advantageous for optimizing patient outcome relative to all-or nothing therapy by surgical myectomy or alcohol ablation.

## Background

Hypertrophic cardiomyopathy (HCM) is a relatively common genetic cardiovascular disease, occurring in more than 1 in 500 people [1, 2]. HCM can occur in several regions of the myocardium, and is particularly troubling when restrictions occur in the left ventricular outflow tract (LVOT). Patients may experience shortness of breath, angina, palpitations, and even sudden death in young athletes [3]. Hypertrophy leads to obstruction of the LVOT in up to 75% of patients, either at rest or with exercise. One-third of patients with obstruction remain symptomatic after pharmacological therapy and are candidates for myocardial reduction [4]. Apical hypertrophy is also troubling and currently has no available corrective therapy [5].

Surgical septal myectomy is presently the primary method for reduction of asymmetric septal hypertrophy with perturbation of the mitral valve leaflets [6, 7]. This invasive procedure simply deletes a volume of the myocardium to relieve the obstruction. For HCM patients with surgical myectomy, the interventricular wall thickness was reduced by 18% from 19.4 to 15.9 mm [8]. Transcatheter septal ablation with alcohol has been developed as an alternative to surgery [7], and has achieved some success in terms of safety and efficacy [9]. The immediate therapeutic benefit appears to depend on akinesis of the effected region, rather than an immediate reduction in size. However, septal thinning develops gradually after alcohol treatment and reductions from 23.7 to 18.0 mm (24%) have been reported after 6 months [10]. The alcohol procedure results in a volume of complete necrosis, which progresses to myocardial fibrosis and scar [11]. This procedure has a high incidence of heart block (about 20%) requiring permanent pacemaker [4], and healing of the alcohol infarction to massive scar carries a risk of serious arrhythmia [12]. These procedures are comparable with low rates in overall mortality and sudden cardiac death after treatment [9].

An alternative to myectomy or alcohol ablation would be a useful additional option for some patients if it were less invasive or more precise in creation of myocardial reduction or akinesis, and could be utilized for apical or other presentations of hypertrophy not suitable for the established treatments. We have developed a novel ultrasonic method of myocardial treatment, which employs intermittent pulsed ultrasound exposure of the myocardium during infusion of a microbubble contrast agent. Infused microbubbles are destroyed by relatively high pressure amplitude pulses, and the intermittent delay allows capillaries to refill with blood containing fresh microbubbles. The microbubble destruction nucleates ultrasonic cavitation within the focal zone, leading to randomly scattered microlesions each with capillary injury and one or a few lethally injured cardiomyocytes. This nonthermal myocardial cavitation-enabled treatment (MCET) method was based on the diagnostic myocardial contrast echocardiography technique using contrast destructive pulses with observation of contrast refill for perfusion assessment. Scattered cardiomyocyte injury was detected for myocardial contrast echocardiography [13], but MCET employs higher than diagnostic pressure amplitudes for therapeutic myocardial reduction [14]. This treatment produces microlesions which accumulate with each intermittent pulse allowing adjustment of microlesion density from modest fractions of tissue volume to more dense distributions within a targeted focal zone [15]. The effected region can be accurately created by the image-guided placement of the ultrasonic focus. Echocardiography is the gold standard method for HCM diagnosis and treatment follow up. The combination of MCET with diagnostic imaging presents a compelling new concept of non-invasive and highly controlled HCM therapy.

In studies with rats, MCET has utilized a small 1.5 MHz focused transducer aimed with guidance from a 10 MHz cardiac probe on a GE Vivid 7 Dimension ultrasound machine [14]. Highly effective treatment involves 10 cycle pulses of 4 MPa peak rarefactional pressure amplitude delivered intermittently each 8 heartbeats during continuous IV infusion of microbubble contrast agent suspension for 10 min of lesion accumulation. Most pulse-triggers produced premature complexes (PCs) in the ECG, when triggered at the end of systole, which are indicative of treatment progress [16]. If pulse triggers produce few PCs, then the resulting treatment effect will be inadequate. Troponin I measurements in blood samples provide an overall measure of treatment impact. Wall thickness and wall strain and displacement are measured by echocardiography, and fibrosis is assessed in histology.

Longer-term maturation of the myocardial volumes with MCET microlesions was studied to assess the potential therapeutic effect in normal Dahl-SS rats [17], and in the SS-16^BN^ rat model of HCM [18]. The consomic Dahl SS rat with chromosome 16 from brown Norway rats develops progressive left ventricular (LV) hypertrophy without hypertension [19]. For normal rats, significant inflammatory response to the injury increased the heart wall thickness the day after treatment, as measured by echocardiography [17]. After 4 weeks, no significant tissue reduction was noted in the treated region with scattered areas of tissue fibrosis. For the HCM model rats, the inflammatory response was greatly reduced by adjuvant steroid given at treatment and adjuvant treatment with an angiotensin II receptor blocker was used to minimize scarring [18]. MCET rapidly produced a significant reduction in myocardial strain rate and endocardial displacement. At 28 d post therapy, the targeted wall thickness was significantly reduced by 16.2% (*p* < 0.01) relative to shams, and strain rate and displacement were, reduced by 34% and 29%, respectively, which are sufficient for therapeutic treatment.

An important advantage of MCET, for example, relative to alcohol ablation, is the ability to monitor outcome by echocardiography, and re-treat desired segments of the myocardium by image guided placement of the focal zone. This is limited by the depth of the focal zone, so that in rats, the 1.5 MHz focal zone passes through the heart giving lesions on the anterior, posterior and septal regions. A system designed for MCET in humans would have the ability to target desired regions more specifically, and to rapidly treat by intermittent scans. Another advantageous feature is the ability to gradually accumulate microlesions to form a desired “macrolesion” treatment impact. A conservative treatment could be augmented at a later date and adjusted in shape and size as desired. This present study was designed to test the idea of multiple treatment accumulation using the normal rat model. Three treatments of 200 s, spaced a week apart, were compared to 3 sham 200 s treatments, one 200 s treatment and one 600 s treatment (i. e., the 10 min highly efficacious protocol).

## Methods

### Animal preparation

All in vivo animal procedures were conducted with the approval and guidance of the University Committee on Use and Care of Animals of the University of Michigan. Normal Dahl SS rats (Charles River, Wilmington, MA, USA) were used for this study for comparison to earlier work [14, 17]. For ultrasound treatment, the rats were anesthetized by intraperitoneal injection of ketamine (90 mg kg^−1^) and xylazine (9 ml kg^−1^) and the left thorax was shaved and depilated for ultrasound transmission to the heart. A 24 gauge cannula (BD Angiocath, Becton Dickinson Infusion Therapy Systems, Sandy, UT, USA) was inserted into the tail vein for intravenous injections of contrast agent. The rats were mounted on a positioning board and needle electrodes were placed in the forelegs and left hind leg for detection of the electrocardiogram (ECG). The board was then placed in a 38 °C water bath filled with degassed water which maintained the rat body temperature and provided coupling for ultrasound scanning and treatment. All rats were allowed to waken and had follow-up monitoring for 6 weeks. For follow-up echocardiography or other exams, the rats were anesthetized with 1–4% isoflurane in oxygen (Isotec 4, Surgivet Inc. Waukesha, WI USA), and scanned in the right lateral recumbent position on a warming pad.

### Ultrasound

Ultrasound exposure for treatment was provided by a laboratory system with guidance by diagnostic ultrasound imaging, as described previously [14]. Briefly, the treatment system consisted of a function generator for generating a train of 10 cycle pulses at 4 kHz PRF (model 3314A function generator, Hewlett Packard Co., Palo Alto CA, USA). An arbitrary waveform generator was used for amplitude modulation of the pulse train to give 8 pulse bursts (model 33220A, Agilent Technologies, Loveland CO, USA). A power amplifier (A-500, Electronic Navigation Industries, Rochester NY, USA) elevated the signal for driving the transducer. The rat ECG signal was amplified (Model ECGA amplifier, Hugo Sachs Elektronik, Harvard Apparatus, March, Germany) and displayed on an oscilloscope (Model TDS 520 B, Tektronix Inc., Beaverton, OR, USA), which was used to trigger the pulse bursts at end of systole. A 1.5 MHz single element transducer (Panametrics A3464, Olympus, Waltham, MA, USA) with 1.9 cm diameter and 3.8 cm focal length was used for the treatment. The pulse exposure parameters were measured with a calibrated hydrophone (model HMA0200 membrane hydrophone, Onda Corp. Sunnyvale CA USA), and used to adjust the function generator voltage to generate a 4 MPa peak rarefactional pressure amplitude (PRPA). The chest wall attenuation at 1.5 MHz is about 1.1 dB/cm/MHz [20], which reduces the PRPA at the heart to about 3.6 MPa after passing through the 5 mm thick chest wall. The equivalent in situ MI (the derated PRPA divided by the square-root of frequency) was therefore about 2.9; for reference, the regulatory upper limit on the MI for diagnostic ultrasound is 1.9. The −6 dB beam diameter was measured at the focus to be 3.5 mm. The focus was targeted to the anterior surface of the heart with the aid of 10 MHz diagnostic ultrasound imaging (GE Vivid 7 with 10S probe, General Electric Corp., Cincinnati OH, USA), as previously described [14]. Treatments were either 200 s or 600 s in duration.

### Ultrasound contrast agent

A continuous infusion of a microbubble suspension was given during treatment. The suspension was prepared to closely duplicate the ultrasound contrast agent Definity® (Lantheus Medical Imaging, Inc., N. Billerica, MA), as described previously [17, 21]. This suspension served as the source of in vivo cavitation nuclei for MCET, and contained 3.7 ± 0.3 (10^9^) microbubbles per mL of 1.8 ± 0.11 μm diameter. This was diluted in sterile saline in a 5 ml syringe, and infused via tail vein with a syringe pump at a rate providing 5 μl/kg/min of agent.

### Adjuvant treatment

Adjuvant treatment with methylprednisolone (methylprednisolone acetate injectable suspension, Depo-Medrol, Pfizer Inc. New York, NY USA) and losartan (Losartan Potassium, TEVA Pharmaceuticals USA, INC. North Wales, PA) was given to the rats to improve the treatment outcome. Roberts et al. showed that initial inflammation and subsequent scarring from myocardial infarction in rats was reduced by high dose methylprednisolone treatment [22]. For MCET, methylprednisolone reduced the inflammatory response to microlesion injury and the swelling of the treated area [18]. Doses of 1 mg/kg were given at treatment and at 4 and 24 h post treatment. The steroid treatment had a negative effect on the weight of the rats, and weights were measured at treatment and each day of follow-up. de Carvalho Frimm et al. reported that losartan, an angiotensin II receptor antagonist used for blood pressure control, reduced infarct size and collagen volume fraction (indicative of scarring) at 4 weeks post infarct [23]. For this study, losartan was given daily by gavage at 10 mg/kg in water, starting 1 day after ultrasound treatment and continuing for until the conclusion of the study.

### Measured endpoints

Plasma troponin was measured in samples obtained 4 h after treatment, which provided a general measure of overall heart injury. Plasma troponin I (CTNI-2-HSP, Rat Cardiac Troponin-1 ELISA kit, Life Diagnostics Inc. West Chester, PA USA) is a sensitive measure of cardiac injury in rodents with essentially zero background in controls [24]. In previous work, the plasma troponin correlated with wall thinning observed in HCM model rats at 4 w post treatment [18]. The cumulative release of troponin is a useful measure of total injury [25].

Left ventricular wall thickness and ejection fraction was measured by echocardiography at treatment (D0), the next day (D1) to assess the inflammatory increase in wall thickness, after 1 week (D7), and at euthanasia. In addition, the local reduction in kinetic motions of heart wall was measured by echocardiography using image analysis software (EchoInsight, Epsilon Imaging, Ann Arbor MI USA) as described previously [18]. The circumferential strain fraction (elasticity) and the displacement of the endocardium from diastole to systole were evaluated. For repeated measurements, the treated region could be identified accurately by the acoustic window and by hyperechogenicity in the ultrasound image (caused by the inflammatory response or fibrosis for later exams). These were compared to matched regions in sham treated hearts.

The healing and scar formation was also evaluated histologically, as described previously [18]. The hearts were fixed in an end-diastolic configuration [26], giving with dimensions approximating the dimensions observed by echocardiography. Histological slides were processed at the Histology Core of the University of Michigan Dental School with Masson’s trichrome staining to show fibrosis. The wall thickness change was estimated relative to shams. In addition, the approximate percentage of the treated areas with fibrosis was assessed by image analysis of blue Masson’s staining.

### Experimental plan

Twenty-four Dahl SS rats, 20–22 weeks of age, were used for this study in 4 groups, listed in Table 1. The groups were: A. sham multiple treatment (5 rats), B. three 200 s treatments (6 rats), C. single 200 s treatment (6 rats) and D. single 600 s treatment (6 rats). One rat died in animal housing before use, which was subtracted from the sham group. One rat from the multi-treatment group died 35 d after beginning treatment, with that final datum lost from the data set. Rats were randomly chosen for each group. Four rats were used each of 12 treatment days, which were selected from the groups in a defined pattern to balance the order of handling in each group. This was complicated by the multiple treatments. Each treatment day, 3 rats received one of their multiple treatments (alternating 1A and 2B with 1B and 2A), and one rat received its single treatment (alternating group C or D).

**Table 1:** Parameters for the rats at initial anesthesia (average ± standard deviation)

			Weight	HR	SpO_2_
Group	Treatment	n	gm	BPM	%
A	3× sham	5	372 ± 18	265 ± 23	82 ± 2
B	3 × 200 s	6	391 ± 12	261 ± 11	90 ± 4
C	200 s	6	405 ± 20	246 ± 14	85 ± 5
D	600 s	6	395 ± 29	253 ± 18	86 ± 4

The groups of rats were very similar, with some variation in weight due to the experimental plan having a staggered start (rats differing in age by up to 3 weeks)

Results are reported as the means plus or minus one standard deviation, or plotted with standard error bars. Statistical analysis was performed (SigmaPlot for Windows V. 11.0, Systat Software Inc., San Jose CA, USA). Student’s *t*-tests were used to compare means of the measured parameters, with statistical significance assumed at *p* < 0.05.

## Results

**Fig. 1:**
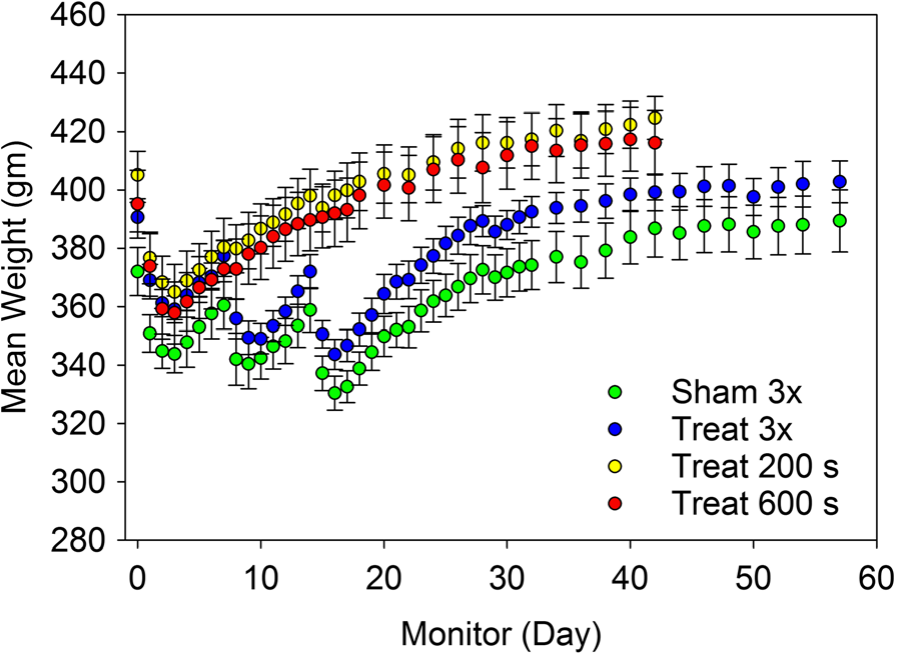
Trends in the mean body weight for each of the groups. The steroid treatment induced a brief period of weight loss for each treatment session so that the fractionated treatment groups, both sham and exposed, had three brief periods of weight loss

The physiological parameters for the groups are listed in Table 1. All had approximately the same weight, heart rate under anesthesia and SpO_2_ under anesthesia. The rats all received the steroid treatment at each exposure session. Therefore the fractionated exposure with three 200 s exposures had 3 dose regimens of the steroid. The steroid treatment produced a brief period of weight reduction in the Dahl SS rats, considered to be an adverse reaction. The weight trends for each group are plotted in Fig. 1. The fractionated treatment groups (sham and exposed) had quite similar trends of repeated brief weight loss, which indicates the weight changes were due to the steroid, and not the MCET treatment.

**Fig. 2:**
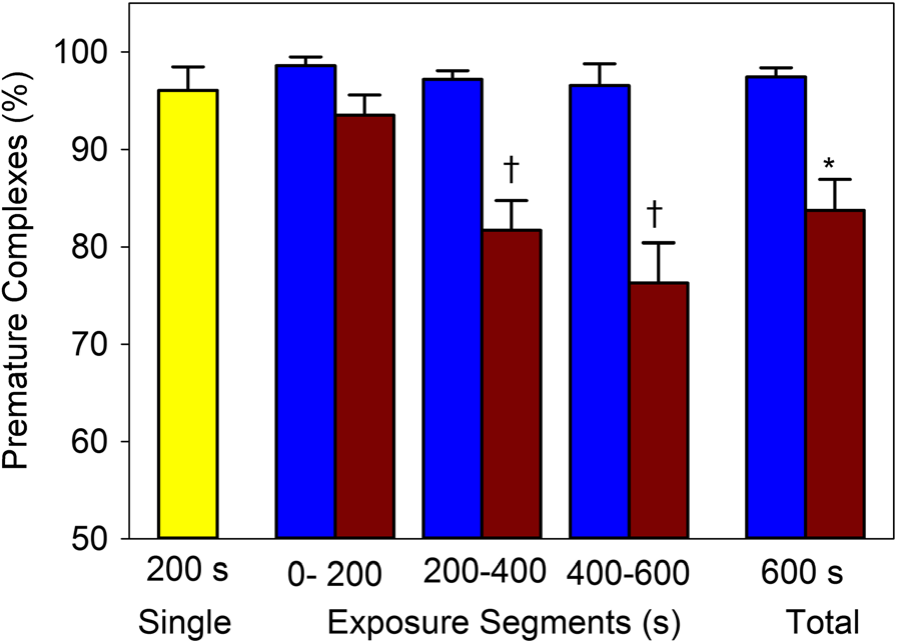
Results for the percentage of pulse triggers resulting in premature complexes in the ECG (average ± standard deviation) for 200 s (yellow), 600 s (red) and fractionated treatment (blue). PCs were significantly less than in the 1st period for the successive periods of the 600 s treatment (†), and the total was significantly less than the total for the fractionated treatments (*)

The results for PCs and troponin release at each treatment are listed in Table 2. The PCs for the 600 s group are broken down into 200 s segments for comparison to the fractionated treatment group, as shown in Fig. 2. For the fractionated treatment groups, the results for the three treatments are also totaled for comparison to the 600 s group. There was a significant trend for the PCs to decline during the 600 s exposures such that the 2nd and 3rd 200 s periods had significantly fewer PCs than the first period, and the total was significantly less than for the fractionated treatment. Interestingly, although PCs remained the same (≥97%), troponin release for the 2nd and 3rd fractions of the fractionated treatment, which were about the same, were significantly less than the first fraction (*p* < 0.01). The troponin total for the fractionated treatment was somewhat less than for the single 600 s treatment, but the difference was not significant (*p* = 0.06).

**Table 2:** Results for the percentage of pulse triggers resulting in premature complexes (PCs) in the ECG, and for the plasma troponin at 4 h (average ± standard deviation)

	Premature Complexes (%)				Plasma Troponin (ng/ml)			
Group	Exp 1	Exp 2	Exp 3	Mean	Exp 1	Exp 2	Exp 3	Total
A	0.2 ± 0.5	0 ± 0	0.4 ± 0.9	0.2 ± 0.5	0.3 ± 0.02	0.3 ± 0.02	0.48 ± 0.03	1.1 ± 0.04
B	99 ± 2	97 ± 2	97 ± 5	97 ± 2	11.1 ± 3.3	5.6 ± 2.0^c^	6.0 ± 1.4^c^	22.7 ± 5.4
C	96 ± 6			96 ± 6	10.5 ± 3.2			10.5 ± 3.2
D	94 ± 5	82 ± 7^a^	76 ± 10^†^	85 ± 10^b^	29.9 ± 6.4			29.9 ± 6.4

PC results for the 600 s treatment were assessed for each 200 s period, for comparison to the fractionated treatments. Results for the multiple treatments were summed to give the total effect. PCs were significantly less than in the 1st period for the successive periods of the 600 s treatment (^a^), and the mean was significantly less than the mean for the fractionated treatments (^b^). Troponin release was significantly reduced for the 2nd and 3rd of the fractionated treatments (^c^)

**Fig. 3:**
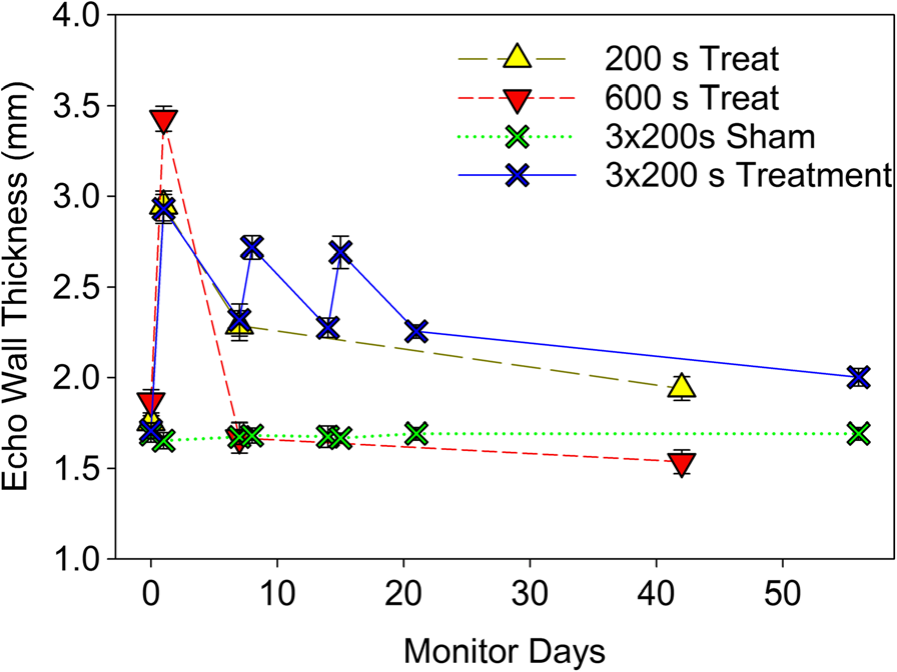
The trends in echocardiographic wall thickness for the groups. Treatment induced immediate swelling which was notable the next day, then receded after 1 week and at the end of monitoring. Only the 600 s treatment gave a significant reduction in wall thickness (*p* < 0.01)

The results of the follow-up monitoring are listed in Table 3 for the baseline and end (i. e. the final exam before sacrifice) echocardiographic wall thickness, ejection fraction, wall strain and endocardial displacement determined from the echocardiographic images, the wall thickness measured in histological samples, and the percentage of fibrotic area seen in Masson’s trichrome slides within the target region. The results were significantly different from baseline for all treated groups. The treatment caused wall swelling after 1 day, which decreased for subsequent measurements at 1 week and at the end of the monitoring. These trends are shown in Fig. 3. For the fractionated treatments, the 3 episodes of swelling and recovery are clearly apparent. Of note, the 600 s treatment group had the wall thickness recede to pre-treatment dimensions, equal to the sham values, after 1 week. The final wall thickness was significantly increased for the 200 s and fractionated treatment, but significantly reduced for the 600 s treatment.

**Table 3:** Results for treatment impact given as the baseline compared to end results for the final exam before sacrifice (mean ± standard deviation)

	Echo wall (mm)		Ejection Fraction		Echo Strain (%)		Displace. (mm)		Histo wall	Fibrosis
Group	Baseline	End	Baseline	End	Baseline	End	Baseline	End	mm	Area %
A	1.7 ± 0.1	1.7 ± 0.1	80 ± 3	77 ± 1	51 ± 7	46 ± 4	0.88 ± 0.13	0.76 ± 0.08	2.3 ± 0.4	0
B	1.7 ± 0.1	2.0 ± 0.1**	79 ± 1	75 ± 2**	45 ± 8	30 ± 5**	0.92 ± 0.06	0.76 ± 0.08*	2.4 ± 0.2	40 ± 10^a^
C	1.8 ± 0.1	1.9 ± 0.2*	80 ± 4	77 ± 5	46 ± 5	36 ± 8*	0.87 ± 0.08	0.84 ± 0.11	2.4 ± 0.4	29 ± 16^a^
D	1.9 ± 0.2	1.5 ± 0.2**	79 ± 3	65 ± 6**	45 ± 7	25 ± 7**	0.88 ± 0.08	0.54 ± 0.11**	1.8 ± 0.3^a^	49 ± 8^a^

Significant differences versus baseline denoted by * for *p* < 0.05 or ** for *p* < 0.01. Significant difference versus sham (A) denoted by ^a^. In addition, the fibrosis was higher in D than in C (*p* < 0.05), but there was no significant differences for the other comparisons (B versus C, and B versus D)

**Fig. 4:**
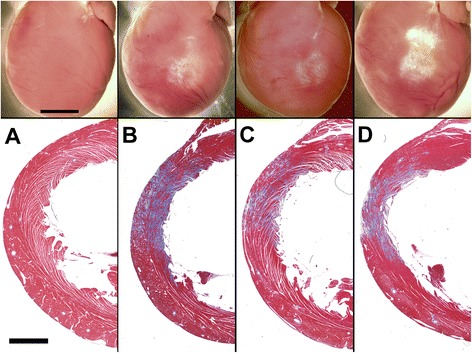
Selected examples of the hearts for each group. Photographs are shown of hearts for groups A-D (scale bar 5 mm), and corresponding histological sections (scale bar 2 mm) stained with Masson’s trichrome to reveal fibrosis (blue). For the 600 s treatment (D), the wall is noticeably thinned in the target area, including some erosion of the heart surface

The heart samples showed visual evidence of fibrosis, as shown in Fig. 4. The fibrosis had a characteristic white circular appearance which corresponded to the beam target area. In addition, for the 600 s groups, an extra region of fibrosis was sometimes clearly evident toward the upper right of the target area; the clearest example is shown in the figure. In histology, the fibrosis, stained blue in Masson’s trichrome histology (Fig. 4), was evident as scattered microlesion regions in the 200 s and fractionated exposures. In the 600 s treatment shown, the section reveals a thin region of tissue erosion on the anterior surface (possibly, giving the very bright white appearance on the heart surface).

## Discussion

MCET produces scattered microlesions encompassing a few lethally injured cardiomyocytes, which accumulate with each triggered pulse burst. The total “macrolesion” can be sufficient for therapy of hypertrophic cardiomyopathy both in terms of heart tissue volume reduction and in akinesis of wall motion and strain. Previous work with Dahl SS rats had demonstrated substantial tissue impact, with minimal impact on heart function after healing for single long (600 s) treatments [17, 18]. Adjuvant treatment with methylprednisolone and losartan reduce the initial swelling and aid in reduction of fibrosis. In this study, the strategy of fractionated treatment which could allow graded treatment and retreatment adjustments was assessed in normal Dahl SS rats. This strategy is an important distinction from surgical myectomy and alcohol trans-catheter ablation, the currently accepted treatments, because those methods are essentially single conclusive treatments.

This study demonstrated that repeated treatments were well tolerated and produced incremental increases in effect as measured by troponin release. However, the 2nd and 3rd treatments a week apart were less effective than the first. The treatment did not produce a reduction in wall thickness, which remained greater than the baseline (Fig. 3). However, the akinesis produced by the fractionated treatment was significant. Akinesis induced by alcohol ablation appears to be responsible for the immediate relief of symptoms [10], and should be therapeutic also for the MCET method.

The single 600 s treatment, used for previous chronic studies [17, 18], was remarkably effective in this study with a 21% reduction in wall thickness (Table 3). This effectiveness may have included a secondary mechanism of injury. The heart shown in Fig. 4 from group D has an exaggerated fibrotic region which appears to be outside the targeted region. For the fractionated treatment, the real time indicator of PCs was the same for the 3 fractions, but declined for each 200 s segment of the 600 s treatment, while apparently yielding greater impact. One possible explanation for the extra fibrosis region is heart motion due to breathing, which periodically could shift the target point of the ultrasound beam. However, the histologically observed result (Fig. 4) seemed to be greater than within the target region, and included a surface layer of myocardial ablation not unlike that seem in ischemia induced infarcts. Thus, another explanation for the high efficacy of the 600 s treatment may be local ischemia resulting from the tissue swelling or microvascular occlusion. This possibility should be investigated and minimized to allow better treatment control for the desired outcome of scattered microlesions within a functional treated volume.

## Conclusions

In conclusion, the strategy of MCET treatment fractionation was shown to be feasible, and represents a potential advantage relative to currently accepted therapy. A possible adverse effect of partial ablation was seen for a 600 s treatment, not previously noted for MCET. However, this was not noted for the fractionated treatment. An unexpected contrast was found for the resulting left ventricular wall thickness, which was increased for fractionated and decreased for continuous treatment. Previously, wall thickness reduction was not seen for normal Dahl SS rats [17] without adjuvant treatment but was seen for the SS-16^BN^ rat model of HCM with the adjuvant treatments [18]. Further study of the safety and efficacy of MCET is needed, especially for larger animal models such as cats [27], which have a more clinically relevant presentation of HCM.
